# Aligning Public Health With a Well-Being Economy: Opportunities and Challenges in Addressing Root Causes of Health Inequities: Comment on "Can a Well-Being Economy Save Us?"

**DOI:** 10.34172/ijhpm.8877

**Published:** 2025-05-18

**Authors:** Lindsay McLaren

**Affiliations:** Department of Community Health Sciences, University of Calgary, Calgary, AB, Canada.

**Keywords:** Well-Being Economy, Political Economy, Public Health, Capitalism, Social Determinants, Ecological Determinants

## Abstract

Labonté offers important critical optimism around the idea of a well-being economy, which is gaining considerable international momentum and offers a much-needed alternative to the current political economic paradigm of neoliberal capitalism and its significant social and ecological consequences. Because of its focus on systems and structures that constitute "root causes" of poor health and health inequities at the population level, a well-being economy aligns strongly with stated tenets and value commitments of public health. It thus provides an important opportunity for public health communities to engage and mobilize as a collective around this important vision. For this to happen, however, public health communities must overcome a reluctance to engage with political economy and take seriously the field’s commitment to the public’s health. In this commentary I reflect on these opportunities and challenges in the Canadian public health context.

## Introduction

 A *well-being economy* is an economy designed to serve all people and the planet, rather than the other way around. In his editorial, Labonté^1^ offers critical optimism around a well-being economy as an alternative to the current political economic paradigm of neoliberal capitalism. He summarizes key efforts internationally to embed well-being economic thinking into policy, which speak to the idea’s growing momentum. He appropriately cautions that the answer to the question, *Can a well-being economy save us?,* has much to do with the extent to which those working to advance such a vision are willing to engage with the obstructive political economic dynamics that fundamentally shape what decisions are made, by whom, and to what ends. In this commentary I consider the well-being economy from a public health perspective in the Canadian context, emphasizing that a well-being economy represents a very significant opportunity for public health communities but one for which we not yet equipped to engage.

## The Strength and Appeal of the Well-Being Economy Idea

 Anchored in a critical stance, Labonté^1^ makes two interrelated points around the importance of the well-being economy idea and why health research and policy communities should take it seriously. First is the beautiful simplicity of the basic idea: rather than treating economic growth as an end in and of itself and pursuing it at all costs, a well-being economy puts our human and planetary needs at the centre of its activities, ensuring that those needs are equally met by default. It thus flips the logic of capitalism—exploitation of people and planet for profit—on its head.^2^ This clarity and simplicity set the stage for broad appeal. Indeed, in a 2023 Canadian conference on the topic, which featured diverse examples of well-being economic thinking ranging from “future generations”-oriented legislation, to participatory budgeting, to the democratizing and decarbonizing potential of public banks, to emancipatory “hyper-localized” solutions such as cooperative worker-owned restaurants and complementary currency, this “subversive” element was highlighted. Although the challenges to realizing a well-being economy are very significant (See more below), there is something about the idea that draws people in, while also having transformative potential.^3^

 Second, Labonté^1^ observes that the well-being economy concept goes further than many other potentially radical concepts and initiatives that have come before in the health space, in terms of naming neoliberal capitalism and engaging in deeper critique of its devastating but inherent social and ecological consequences. It is worth re-iterating the point made by critical public health scholars, that even the (otherwise) hard-hitting 2008 final report of the World Health Organization (WHO) Commission on Social Determinants of Health, which named “poor social policies and programs, unfair economic arrangements, and bad politics” as root causes of health inequities,^4^ was silent on the issue of the economic growth paradigm, despite its incompatibility with the Commission’s own goals and recommendations to “close the [equity] gap in a generation.”^5^

 These two observations, namely the broad appeal and the radical orientation, underscore the well-being economy’s important potential for broad civil society activism, which Labonté^1^ notes is essential to any transformative social change. Because of the well-being economy’s focus on the political economic paradigm, which is the common denominator underpinning major economic, political, and health challenges, it offers a common frame to connect progressive voices in diverse and intersecting policy spaces including existing social, economic, and ecological social movements.^6^

 Labonté^1^ points out that “In many ways, the rise of the idea of well-being economies is *déjà vu* for activist public health movements …” (p. 3), including health promotion’s longstanding emphasis on community empowerment. This suggests an important opportunity for public health communities to mobilize around the well-being economy idea and vision. It begs the question, are public health communities ready for such an opportunity?

## Well-Being Economies – Opportunities and Challenges for Public Health

 There is considerable overlap between the stated tenets of public health, and the idea of a well-being economy. As a broad field of research and scholarly inquiry, policy and practice, and activism, public health is concerned with factors shaping health and its inequitable distribution in populations. These include “upstream” social and ecological determinants of health, which can be strengthened via “organized efforts of society” – a phrase common to many definitions of public health.^7^

 As highlighted by scholars working at the intersection of public health and heterodox economics,^8^ a well-being economy, by foregrounding the social and ecological consequences of neoliberal capitalism and offering an alternative paradigm that puts well-being of all people and planet first, aligns with these elements of public health. Raworth’s doughnut economics model provides an illustration.^9^ The “doughnut” illustrates the dual imperatives of ensuring that no one is left behind when it comes to the essentials of life; that is, the *social determinants of health, *including food, housing, high-quality public services, political voice; this is the inner ring of the doughnut; while not exceeding the planet’s life-supporting systems on which we collectively depend – the *ecological determinants of health*; the outer ring of the doughnut.^10^

 Obstacles to the realization of a well-being economy vision are significant and include the immense concentration of wealth and power that is inherent to neoliberal capitalism, which shapes our relationships with one another and our institutions so that they benefit—in ways that are largely and deliberately obscured—a privileged minority rather than the broader public and planet.^11^ A key question is thus whether public health communities are willing to engage deeply with matters of the economy, and with capitalism specifically, because grappling with the reality of current power structures is a pre-requisite for envisioning an alternative distribution of power.

 A recent analysis by McLaren and Mykhalovskiy^12^ sheds some light on this question in the Canadian context. We considered the extent and nature of the Canadian public health community’s historical engagement with economics and economic policy, as gleaned through the pages of our longstanding national journal, the *Canadian Journal of Public Health *(CJPH). Despite the well-established connections between political economic policy, population well-being, and health equity, we noted that current engagement by mainstream public health communities with economic policy tends to be narrow, focusing on issues like the “return on investment” of public health interventions. The analysis thus considered the historical foundation for contemporary public health engagement with economic policy including constructive engagement towards an economic system, such as a well-being economy, that supports, rather than obstructs, population well-being and health equity.

 Based on an in-depth analysis of six historical volumes of the CJPH, each one randomly selected from a period corresponding to key economic circumstances or events^[1]^, we drew three key conclusions. First, we found only a slim historical foundation for public health engagement with the economy overall. Second, we observed a strong and seemingly sub-conscious allegiance to dominant (capitalist) economic paradigms, despite their incompatibility with root causes of health inequities. Third, even though socio-economic inequalities in health are a longstanding preoccupation of CJPH authors (and public health communities more broadly), those inequalities are consistently divorced from their roots in political economic systems.^12^

 Notably, there were some important examples of thoughtful and critical engagement by CJPH authors with matters of the economy, which we selected for accompanying republication in the hopes that they could inspire future reflection and engagement. However, these exemplars were sparse and tended to be limited to commentaries rather than integrated into empirical research. Overall, our analysis suggested that, at least in terms of the CJPH historical record and its Canadian context, public health actors, including researchers and practitioners, are ill equipped to seriously engage with economic policy issues^[2]^. The continued dominance of implicit and explicit economic liberalism and residualism in the field of public health, which is intertwined with the field’s medical origins, serves to obstruct—we argued—the community’s ability to work coherently towards its own stated goals of population well-being and health equity, which are fundamentally rooted in political economic and other intersecting systems.

 More recent, anecdotal, examples in the Canadian context support the contention that we still have considerable work to do. In 2022, in the wake of the COVID-19 pandemic, the Canadian Institutes of Health Research (Canada’s federal health research funding agency) invited applications to a funding competition focused on “Transforming Public Health” including via “upstream interventions that address the social determinants of health and have the potential for significant impact across multiple public health priorities.”^13^ An interdisciplinary group of colleagues and I developed and submitted a proposal titled “Transforming public health: building critical foundations for a well-being economy,” where we argued that, because the root causes of poor health and health inequities lie in dominant political economic systems and the economic growth paradigm, any vision to transform public health that does not meaningfully engage with those root causes is incomplete. We offered the well-being economy idea as a frame to advance those conversations.

 Illustrative of the field’s inability, or unwillingness, to see the connections between political economy and the public’s health, our application was disqualified as “non-relevant.”^[3]^

 Even more recently, and in the context of growing international interest and momentum around the notion of a well-being economy (which Labonté^1^ helpfully summarizes), public health actors at the federal level in Canada, such as the Chief Public Health Office, have shown interest in the well-being economy concept itself. I have participated in some of these national discussions, and to my dismay—if not surprise—I have observed a shift in language from “well-being economy” to simply “well-being”; below is an illustrative example (excerpt from personal communication, email, June 4, 2024):

 “*The concept of wellbeing – and its applications in metrics, frameworks, and policy making – has been used across sectors as a way to think and act more broadly to achieve shared goals. This event is intended to explore how wellbeing approaches and frameworks can be used to advance public health priorities in Canada. This will include a discussion of existing wellbeing initiatives in Canada, to learn what approaches have had success in bringing sectors together to improve the conditions for health and wellbeing in communities. Invitees will include leaders and experts in public health and health equity, as well as a select number of experts in wellbeing interventions and initiatives.”*

 As noted by Labonté,^1^ a key strength of “well-being economy” is that it puts the two terms (well-being and economy) together as inseparable concepts. It pushes us to engage with the economy and its inherently political dynamics. Omitting “economy” allows us to avoid that crucial part of the discussion, which in turn risks undermining the concept’s radical potential to redress health inequities and improve the public’s health.

 What would it take for mainstream public health communities to engage more meaningfully with a well-being economy as an alternative political economic paradigm? As a starting point, it requires a willingness to engage with deep but rarely acknowledged epistemological divisions and power dynamics within our field.^14^ It also requires having the humility to recognize the serious constraints posed by our usual ways of thinking and working, where we seek “evidence” (itself a term that needs to be problematized) on “interventions” that are conceptualized as singular and discrete. One tangible starting point is to better integrate criticality into post-graduate public health education,^7^ so that incoming public health workers are equipped and empowered to both *look outward*, to uncover and challenge the health damaging effects of particular social structures in their political and historical contexts, and to *look inward*, to question assumptions and illuminate ways in which we and our work are complicit in the political economic status quo.^15^

## Conclusions

 The dilution or sanitizing of potentially radical ideas is nothing new. Indeed, we have seen this occur with other concepts like the social determinants of health and health equity.^16^

 What is perhaps different now is that we are at even more of a crossroads. The current polycrisis—a combination of significant and interconnected problems including the climate emergency, widening inequalities, and ideological extremism^17^—is fundamentally caused by our current political economic paradigm premised in competition, scarcity, and suffering, and it is worsening in front of our eyes with the political rise of far-right governments. This must prompt us to find ways to engage as a collective to support the public’s health. We can passively await the impending political economy of authoritarian capitalism or worse, or we can try to meaningfully engage with a vision of an alternative—such as a well-being economy—that centres all people, all living things, and our planet through premises such as solidarity, co-operation, respect, and humility.^11^ Public health communities have an important decision to make.

## Ethical issues

 Not applicable.

## Conflicts of interest

 Author declares that she has no conflicts of interest.

## Endnotes


^[1]^ These periods were: 1910-1928, from the journal’s first issue through the first world war and its aftermath; 1929-1938, the 1929 stock market crash and ensuing Great Depression; 1939-1945, the economic preoccupation of the second world war; 1946-1979, the Keynesian period; 1980-2007, the early neoliberal period; and 2008-2021, which Canadian progressive economist Lars Osberg refers to as “zombie neoliberalism” because, while the credibility of neoliberalism has been shattered, an alternative paradigm has not yet emerged.
^[2]^ It would be valuable to have similar analyses of longstanding national public health journals in different jurisdictions.
^[3]^ We appealed the disqualification and were admitted back into the competition, where we were unsuccessful following peer review. It is the disqualification to which I wish to draw attention here, as illustrative of the narrow frame of mainstream public health, at least in the Canadian context.
